# Going beyond “With a Partner” and “Intercourse”: Does Anything Else Influence Sexual Satisfaction among Women? The Sexual Satisfaction Comprehensive Index

**DOI:** 10.3390/ijerph191610232

**Published:** 2022-08-17

**Authors:** Adelaida I. Ogallar-Blanco, Raquel Lara-Moreno, Débora Godoy-Izquierdo

**Affiliations:** 1 Health Psychology and Behavioral Medicine Research Group (CTS-267), Mind, Brain and Behavior Research Center (CIMCYC), University of Granada, Cartuja University Campus, 18071 Granada, Spain; 2 Department of Personality, Evaluation and Psychological Treatment, Faculty of Psychology, University of Granada, Cartuja University Campus, 18071 Granada, Spain; 3 Department of Social Psychology, Faculty of Psychology, University of Granada, Cartuja University Campus, 18071 Granada, Spain

**Keywords:** sexual satisfaction, sexual health, females’ sexuality, personal satisfaction, psychometrics, validation

## Abstract

The validated tools for measuring sexual satisfaction (SS) assess in fact other phenomena such as sexual functioning, assess SS within the context of a romantic heterosexual relationship and intercourse-type activity or were designed to be applied only in therapeutic or rehabilitation contexts. Consequently, they offer an incomplete understanding of SS, particularly among women. We thus developed an alternative measure of SS, the Sexual Satisfaction Comprehensive Index (SSCI), considering both the individual and with-a-partner dimensions, as well as the actual and desired experiences of satisfaction, along with other dimensions affecting SS, and explored its psychometric properties. A total of 1080 young to mid-aged women with different sociodemographic, relationship and sexual identity backgrounds voluntarily completed several measures of SS, including the SSCI. Results showed that the SSCI is a reliable measure for assessing SS. An exploratory and a confirmatory factor analysis confirmed the latent multidimensional structure of the SSCI, supporting its factorial validity. In addition, the SSCI showed appropriate convergent validity with other measures commonly used to assess SS. In sum, the SSCI was revealed to be a useful multidimensional index of SS for research and practice contexts which allows the practitioner or the researcher to make decisions on which dimension(s) are to be evaluated. This study focused on women’s SS, and future research with other gender, sexual and cultural identities is necessary to support its utility in multi-identity and multi-sexuality scenarios.

## 1. Introduction

Sexuality is an essential area of the human experience involving biological, cognitive, emotional, behavioral and social factors, and it is closely related to overall health and quality of life [1,2]. Therefore, it is important to investigate which variables can foster positive aspects of sexuality such as sexual quality, sexual well-being, sexual pleasure or sexual satisfaction (SS), especially in people without dysfunctions or problems, and particularly in women, whose sexuality and sexual pleasure have traditionally been neglected, silenced or punished due to sociocultural factors and patriarchal, male-centered ideologies [3].

Despite the growing interest in studying SS, an agreement has not yet been reached neither on its definition nor its assessment. The fact that it is, both as a construct and as an experience, influenced by individual, social and cultural factors makes it difficult not only to find a consensual definition of the term, but also a consensual instrument to measure it [4]. For the purpose of this study, we define SS as the subjective global experience resulting from the individual’s evaluation of the extent to which the individual and with-a-partner(s) personal needs, desires and expectations regarding sexuality are accomplished through sex and sexuality, which leads to a variety of multi-faceted experiences of pleasure, joy, well-being or happiness at the physical, emotional and cognitive levels. An appropriate or successful sexual functioning, as well as the mere absence of dysfunctions, is, therefore, not enough to guarantee SS, which includes positive experiences of gratification, contentment and fulfilment. This definition is in line with the World Association of Sexual Health (WAS) Declaration of Sexual Pleasure, and the guidance to research and practice inspired by it [5].

Some of the most well-known validated measures for assessing SS in women are the Derogatis Sexual Functioning Inventory (DSFI) [6], the Golombok–Rust Inventory of Sexual Satisfaction (GRISS) [7], the Brief Index of Sexual Functioning for Women (BISF-W) [8], the Female Sexual Function Index (FSFI) [9], the Index of Sexual Satisfaction (ISS) [10], the Global Measure of Sexual Satisfaction (G-MEX) [11], the Sexual Satisfaction Scale–Women (SSS-W) [12] and the New Sexual Satisfaction Scale (NSSS) [13]. For all of them, appropriate psychometric properties have been reported and they have been widely used to assess SS in research and clinical contexts.

Although all of them were designed or are frequently used with the intention to approach SS [14], there are some notable differences between them. Specifically, some (e.g., DSFI, GRISS, BISF-W) assess in fact sexual functioning. Research has related both constructs, and it has been understood that the absence of sexual functioning issues automatically means the presence of SS [15], equaling adequate, expected, positive sexual functioning to sexual well-being. Other tools assess SS within the context of a certain theory (e.g., the interpersonal exchange model of SS in the case of the G-MEX), often considering SS only within the context of a romantic relationship—generally understood as heterosexual (e.g., G-MEX, GRISS, ISS)—as if their creators were only interested in or equaled marital/partnership satisfaction with SS. Others have been developed to be applied within rehabilitation or therapeutic contexts to assess the presence of sexual dysfunctions or using samples with sexual problems (e.g., GRISS, BISF-W, ISS, SSS-W), in order to know to what extent SS is affected by sexual impairments or promoted after sexual therapy.

In addition, all the measures, even those assessing sexual functioning, have included items to assess variables related to SS. For instance, the subjective perception of the participant’s own functioning (DSFI); the frequency of certain sexual behaviors or the communication with the partner (GRISS); body image, sexual anxiety and satisfaction with the partner (BISF-W); whether sexual relationships with the partner have become a chore to the participant and perceived quality (ISS); value and pleasantness attributed to the sexual relationship with the partner (G-MEX); compatibility and interest in the relationship (SSS-W); and sensations, consciousness or concentration during sexual behaviors (NSSS), to name some examples.

Although both one’s own and other’s/others’ sexual functioning and the factors related to a relationship with a partner(s) and its perceived quality have undoubtedly a great impact on SS [16,17,18], we understand that they do not properly refer to SS. In addition, research to date ignores other processes with an influence on SS that might also have an important role. On the one hand, the study of factors related to SS has not often been carried out with the purpose of exploring individual SS itself; instead, research has focused on establishing their influence on sexuality with a partner, e.g., conflict frequency [19], relationship satisfaction [20,21], sexual communication [22], intimacy [23,24], sexual frequency and marital satisfaction [25]. On the other hand, when a partner is considered, almost all the measures consider heterosexual (two-person) couples and intercourse [26]. In addition, the existent self-reports do not differentiate between actual SS, which is usually the focus, and desired or expected SS, and respondents are often confused or find it difficult to separate both experiences [27]. Finally, the available measures do not consider the factors which people think are related to or affect their subjective SS, e.g., the importance given to a healthy or active sexuality, the used techniques or activities or perceived sexual skills, nor the behaviors that lead (or do not lead) to it [14,28,29].

Considering the strengths and limitations commented above for the most used indicators of SS, the aim of this study was to validate an alternative measure of SS that considers both the individual and the with-a-partner dimensions of SS, as well as the actual and the desired experiences of satisfaction related to sex and sexuality. We thus developed, through a multi-stage process, a comprehensive self-report of SS, the Sexual Satisfaction Comprehensive Index, and explored its reliability and validity in a wide sample of Spanish women. Additionally, we explored the SS of these young and adult women and the factors and actions that contribute to participants’ SS, going beyond “a partner” and “sexual intercourse” activities. Our aims will not only allow a more appropriate assessment of women’s SS but also will help to understand the broad and varied predictors of a healthy and satisfactory sexuality among women, with implications for both research and practice/clinical contexts.

## 2. Materials and Methods

### 2.1. Participants

An initial nation-wide sample (N = 1877) of potential participants was obtained through non-probabilistic sampling including individuals who voluntarily accessed the online survey described in the Procedure section. Of these, 11 cases were removed from the database according to methodological criteria (i.e., test cases, duplicates). In addition, 512 individuals did not complete the survey and thus left unanswered the main variables of the study. Therefore, they were also removed. Another 274 cases were discarded because they did not meet the inclusion criteria of this study, i.e., self-identified female gender, age between 18 and 50 years old and having been born or lived in Spain for at least 1 year. Specifically, 169 men who accessed the survey, five women younger than 18 year old and 25 women over the age of 50, one woman not indicating her age, and 74 foreigners who had lived in Spain for less than 1 year were discarded. We intentionally selected Spanish women between 18 (legal age of majority) and 50 years of age because those are the cohorts that have experienced the different social, cultural and political developments derived from the establishment of democracy in Spain [30] and they were not peri- or post-menopausal women [31,32], these being key factors influencing females’ sexuality.

Thus, 1080 women aged 18–50 years (M = 24.48, SD = 6.11) from all over the country participated in this study. Of these women, 1050 (97.2%) were Spanish citizens who had lived in Spain for their whole lives, whereas 30 came from different European (1.8%), Latin-American (0.8%) or Asian countries (0.2%); 94.3 % of them had lived in Spain all their lives, 1.8% for more than 20 years, 2.5% between 11 and 20 years, 0.6% between 6 and 10 years and only 0.8% had lived in Spain between 1 and 5 years. The foreigners finally included in the sample reported having lived in Spain their whole or most part of their lives (98.6%), so cultural variability influences were expected to be minimal. Regarding participants’ sexual orientation, 80.3% considered themselves heterosexual, 13.2% bisexual, 3.1% lesbian and 3.4% reported that they had not yet fully defined their sexual orientation. In addition, 31.1% of the participants indicated that they had no sexual partner at the time of the study, 22.5% had been in a relationship for more than 5 years, 29.9% had been in a relationship between 1 and 5 years and 16.5% had been in a relationship during a year or less. Other characteristics of the participants refer to their education level, religious beliefs, political position and sexual partners (Table 1).

### 2.2. Measure

We developed a comprehensive measure of SS based both on the validated, published above-mentioned tools and on a previously semi-structured interview conducted with women [30], attempting to create a tool which was useful for assessing the multidimensional nature of SS and contributing factors, in both practice and research contexts.

For that, the first step was an in-depth revision of the literature and measures regarding SS. Based on the information obtained, we developed a semi-structured interview that was revised and enriched by researchers and experts in human sexuality before it was applied to a pilot sample of young healthy women. Once this initial version of the interview was updated it was applied to a sample of 52 women [30].

The second step was to analyze the answers to the open-response questions included in the interview in order to group them into categories and frequency. Once the most relevant and representative responses were selected, they were used for formulating the items and alternatives of response of a self-report measure, which was then also reviewed by a number of experts in a Delphi group. Based on their feedback, some items/responses were reformulated. After this, it was applied to a new pilot sample of 15 healthy women for revision and correction.

Lastly, the subsequent reviewed version served to create the definitive self-report measure, the Sexual Satisfaction Comprehensive Index (SSCI) (See Appendix A for the originally applied version in Spanish along with its translation to English). This final version includes 13 questions, all of them assessed on a Likert-type scale. The first four items assess the subjective levels of SS: actual and desired, both for sexual activities conducted individually and with a partner, i.e., Actual With the Partner(s), Actual Individual, Desired With the Partner(s) and Desired Individual satisfaction dimensions (0 = Not satisfactory at all/Not interested in it, 3 = Very satisfactory). Moreover, four additional questions are formulated to assess orgasm-related aspects: its frequency in both individual and interactive sexual activities (0 = I have never reached an orgasm, 3 = Almost always or always); the importance given to achieving an orgasm during both shared and individual sexual activity (0 = No, I fully enjoy my practices without having an orgasm; 4 = Yes, I usually only feel fully satisfied if I manage to have more than one orgasm); and the overall satisfaction achieved during shared or individual sexual activity when orgasm is reached (0 = I usually don’t have orgasms, so I enjoy my practices without orgasms, 5 = I only feel completely satisfied if I manage to have at least one orgasm). Two items assess desire and excitement levels (0 = Null, 4 = Very high). These last six additional items were included to obtain more information on sexual functioning, orgasms and perceived value attributed to orgasm in relation to SS. One additional item includes 32 factors that can play a role in SS, and the participant is asked to rate the level of influence in her personal case (0 = It does not affect me at all, 3 = It affects me a lot). The included factors are specific individual and relational aspects with an influence on sexuality (i.e., sexuality factors). One further item assesses the level of SS achieved when performing a wide variety (namely, 25) of different sexual behaviors (both individual and with-a-partner(s)) (0 = I do not practice it; 1 = Not satisfactory at all, 4 = Very satisfactory). These two last items were added with the intention of gathering information surrounding aspects, behaviors and activities contributing to the respondent’s sexual pleasure. A last question assesses the importance personally given to having an active/agentic and satisfactory sexual life (0 = Not important at all, 3 = Very important).

The online survey also included the validated Spanish versions of other measures, in order to analyze the psychometric properties—convergent validity—of the SSCI. Specifically, we included the Index of Sexual Satisfaction (ISS) [10,33], which includes 25 items in a 0-to-7 Likert scale assessing with-a-partner SS, such as whether the partner enjoys their sexual life, its quality or the satisfaction with the partner’s sexual preferences (Cronbach’s *alpha* = 0.92 in the present study); the New Sexual Satisfaction Scale-Short Form (NSSS-S) [13,34], which assesses SS levels in a 1-to-5 Likert scale regarding 12 different aspects related to satisfaction, such as disinhibition, one’s own body’s sexual functioning, partner’s ability to orgasm or the frequency of her/his sexual activity (Cronbach’s *alpha* = 0.91 in the present study); and the three items of SS of the Female Sexual Function Index (FSFI) [9,35], assessing satisfaction with the amount of emotional closeness and sexual relationship with the partner, and with overall sex life (Cronbach’s *alpha* = 0.96 in the present study). All the Spanish versions have informed of appropriate psychometric properties [33,34,35].

We also included a socio-demographic form and questions regarding personal experiences with sexual partners and sexuality (see Participants and Table 1).

### 2.3. Procedure

An online version of the assessment protocol was created on the Limesurvey^®^ platform (LimeSurvey GmbH, Hamburg, Germany). This survey was publicized through online media (e.g., social networks of Psychology and Sexology professionals, specialized web pages, institutional e-mailing, social forums) and traditional media (e.g., direct requests to students from different Spanish universities to collaborate participating and/or sharing the survey with other women aged 18 yr. and older) in order to recruit a nation-wide large sample.

On the first page of the online survey, the participants were informed about the study, their rights as participants, the confidentiality of their responses and the exclusive use for scientific purposes. Once they had given their consent after reading this information, the participants accessed the measures. They were then broadly informed about the content of the measures and how to complete them. During the procedure, they could choose to answer the whole survey at one time or save their responses and continue with the procedure later, accessing with a personal code. The participants received no compensation or feedback for their responses. Data were gathered from January 2015 to December 2021. Then, the database was downloaded and checked for depuration according to inclusion criteria and for conducting the analyses.

This research was approved by the ethics committee of the authors’ institution in May, 2011. All procedures used in this study adhere to the principles of the 1975 Declaration of Helsinki, revised in 2013.

### 2.4. Study Design and Data Analyses

This is a correlational study with a cross-sectional design with psychometric-validation purposes. Statistical analyses were conducted using SPSS 28 (Statistical Package for Social Sciences; SPSS BMI Inc., Chicago, IL, USA) and AMOS 22 (BMI, SPSS). Preliminary and exploratory data analyses were conducted to detect (and correct) possible errors in data entry, missing data or univariate and multivariate outliers as well as to check data assumptions. No severe univariate or multivariate outliers were detected, and thus all participants were included in the analyses. Missing data were treated with listwise deletion in each analysis. Descriptive analyses (mean and standard deviation for continuous variables, and n and percentages for categorical variables) were conducted. Reliability was analyzed by means of internal consistency by using Cronbach’s *alpha*. In addition, an Exploratory Factor Analysis (EFA) and a Confirmatory Factor Analysis (CFA) were conducted to explore the factorial validity of the SSCI. Factor analysis is a multivariate technique to uncover latent constructs from observed variables [32]. The minimum amount of data for factor analysis was satisfied, with a final sample of 1080, providing a ratio of over 16 cases per variable.

For the EFA, Maximum Likelihood Estimation (MLE) method with Varimax rotation of the matrix of loadings to obtain orthogonal (independent) factors was conducted, after checking recognized criteria for factorability, namely that data meet assumptions of univariate and multivariate normality, homoscedasticity, independence of sampling, linearity, no collinearity and multicollinearity and full level of response, and that items were factorable, i.e., Kaiser–Meyer–Olkin test of sampling adequacy above the commonly recommended value of 0.60 and Barlett’s test of sphericity of identity matrix significant at *p* < 0.05. As our primary goal was to identify latent constructs underlying measured variables, we selected MLE as factor extraction technique [36]. This procedure is more adequate when the goal is to factorize and define a simpler structure of data due to the separation of common and unique variances, even when the total variance explained is often smaller compared to other methods. Yet the criterion of item loadings > 0.30 or > 0.40 is usually recommended for interpreting the resulting factors [37], factor loadings > 0.20 were also considered as a preliminary attempt to gain understanding of underlying latent variables at the item level [38]. The rationale is that a loading is considered significant over a certain threshold depending on the sample size needed for significance, and given the N in the present study, 0.20 could be considered a correct cutoff [39,40].

CFA is often the analytic tool of choice for developing and refining measurement instruments [41]. The CFA is used to determine factors and factor loadings of measured variables to confirm pre-established theoretical structure based on the EFA findings [42]. CFA is appropriate because it “is generally used to *test* [emphasis in original] theory when the analyst has sufficiently strong rationale regarding what factors should be in the data and what variables should define each factor” ([43], p. 395). Thus, CFA allowed us the exploration of the relationship between observed variables (i.e., SSCI scores) and latent variables or factors (i.e., the dimensions assessed by the SSCI), helping in assessing construct validity. CFA was conducted with Structural Equation Modeling (SEM), with Maximum Likelihood (ML) method (once the above-mentioned distributional assumptions were confirmed) [39,44]. Standardized structure coefficients were reported. Following recent recommendations [41,44], the Chi square goodness of fit test, one incremental fit index: the Comparative Fit Index (CFI) and one residuals-based fit index: the Root Mean Square Error of Approximation (RMSEA), were calculated as goodness of fit indices. Recommended cutoffs of 0.95 for incremental fit indices and of 0.06 for residuals-based indices were adopted [45]. A model was determined to exhibit “good” (i.e., all three fit indices meeting the minimum threshold for fit), “marginal” (i.e., any two of the three fit indices meeting the minimum threshold for fit) or “poor” (i.e., at least two fit indices failing to exceed the minimum threshold for fit) model-data fit based on the comparisons [46].

Finally, convergent construct validity with three well-known measures used to assess SS was established by order-zero Pearson’s correlation analyses.

## 3. Results

Table 2 shows the descriptive results obtained for all the variables. All measures of SS had high mean scores and low standard deviations, showing considerable homogeneity among the participants. Of SSCI satisfaction dimensions, Actual SS levels were lower than Desired SS levels, having Desired With the Partner(s) SS the highest mean and the lowest standard deviation of all dimensions and Actual Individual SS the lowest mean. Regarding the rest of the SSCI variables, all showed high means and low standard deviations. Orgasm frequency, importance and satisfaction were high, whereas Desire and Excitement Levels were moderate and more homogeneously distributed. The importance conceded to sexuality by the participants was notable. Finally, mean values for ISS, NSSS-S and FSFI were high and showed little heterogeneity.

Sexuality factors, behavioral factors and sexual functioning factors deserve a more detailed exploration. Hence, frequency analyses for categorical variables were conducted, i.e., sexuality factors (Table 3) and sexual behaviors (Table 4). The features the participants rated as those “more likely to affect” their SS were: feeling attracted to the sexual partner (72.5%), not feeling obliged to do something not wanted/desired (72.1%), having confidence with the sexual partner (66.8%), experiencing sexuality as something normal/healthy/desirable (65.5%), desiring to have a sexual relationship (63.8%) and feeling comfortable with one’s own body/not feeling ashamed (63.3%). On the contrary, the use of fantasies, role-playing or erotic toys (8.3%), using this or that contraceptive method (14%) or feeling that the sexual partner is committed to the relationship (14%) were the factors rated as having less impact on SS. Indeed, love (e.g., item 19) and sharing (e.g., items 24 and 29) factors, along with orgasm factors (e.g., item 23) obtained low percentages of agreement response.

Sexual behaviors rated as most satisfying were: cuddling (78.5%), kissing (77.6%), hugging (71.9%), vaginal intercourse (70.6%) and licking/biting the sexual partner and being licked/bitten by the sexual partner (62.7%). Those rated as less satisfying, but also as less frequently performed, were: engaging in potentially risky sexual practices (0.9% and 90.8%, respectively), engaging in aggressive sexual practices (e.g., Bondage-Discipline-Domination-Sadism-Masochism) (3.1% and 82.9%), engaging in sex with more than one sexual partner (e.g., threesomes, swinging, orgies, dogging) (4.8% and 82%), engaging in sexual practices aimed to delay climax (e.g., tantric sex) (7% and 66.2%), giving or receiving anal oral sex (9.6% and 63.6%) and anal intercourse (3.5% and 62.3%).

Regarding orgasms and sexual functioning questions, forty-three percent of the participants reported reaching orgasm always or almost always during sexual practices with a partner, whereas 59.5% reported climax during individual sexual practices. On the other hand, almost twice the participants (13.6%) reported having not reached an orgasm during their individual sexual practices versus with-a-partner activities (7.4%). Twenty-two percent reported reaching orgasm sometimes during with-a-partner(s) activities and 27.5% quite a few times, while for individual activities frequencies were lower (14.3% and 12.6%, respectively). Regarding the importance given to reaching climax, both in shared or individual sexual practices, 58.3% reported that orgasm would make their sexual practices more enjoyable, but that it was not strictly necessary, 5.5% would feel more satisfied if reaching more than one orgasm and 29.8% would require at least one to feel satisfied; contrarily only 3% reported that they really do not care about orgasms, and 3.5% affirmed that they enjoyed their sexual activities without reaching climax. As for the overall SS related to orgasm, 9.8% would feel completely satisfied only if reaching at least one orgasm, although 35.8%, 29.3% and 14.3% report that orgasm would make their sexual activities “quite”, “very” and “somewhat more satisfactory”, respectively. For 5.6%, reaching an orgasm would not make any difference, while 5.2% reported enjoying their sexual activities without orgasms. Desire and arousal level were rather similar, rated as “very high” by 25% and 22.8% of the participants respectively, and as “high” by 46.9% and 61%, “moderate” by 26.4% and 12.3% and “very low” or “null” by 1.7% and 4%, respectively. Finally, for 35.6% of the participants, having an active and satisfying sexuality was very important, and considerably important for another 47.6% of the women, while only 16.3% considered it not very important, and only 0.5% though it was not important at all.

Pearson correlation analyses (Table 5) indicated that all the four dimensions of SS assessed by the SSCI were positively correlated with each other, with the exception of Actual Individual SS and Desired with the Partner(s) SS. In addition, the four dimensions were correlated with almost all the indicators surrounding orgasm, sexual functioning and importance conceded to sexuality, which were significantly intercorrelated. Moreover, Actual With-a-Partner SS was the dimension showing the highest associations with the ISS, the NSSS-S and the FSFI, whereas the remaining dimensions showed in general significant yet lower values. Finally, higher correlations were in general found between the ISS, the NSSS-S and the FSFI.

Finally, factorial validity was tested. As a first step, an EFA was conducted with MLE method for factor extraction and Varimax rotation of the factor loading matrix. A Kaiser–Meyer–Olkin index of 0.77, Barlett’s sphericity Chi-Square = 7460.968, *p* = 0.000 and Goodness of fit (Chi-Square = 4014.998, *p* = 0.000) indicated the appropriateness of the EFA. In addition, the communalities found for all the items were appropriate (all values between 0.4 and 0.8, excepting Desired With-a-Partner SS = 0.27 and Sexual Factors item 18 = 0.37; given these values, we decided to initially retain all the items for the EFA). We conducted both an orthogonal Varimax rotation and an oblique Oblimin rotation; given the absolute values of factor correlations between 0.02 and 0.22 we opted for Varimax rotation for the final solution. Furthermore, the eigenvalue-based initial solution was hardly meaningful; thus, we forced a four-factor solution, which was supported by (a) the leveling off of eigen values on the scree plot after four factors, as well as (b) the number of primary loadings in each factor and the difficulty of interpreting the five-factor solution and subsequent ones. Thus, the four-factor solution was preferred because of its support for the multidimensional structure of the SSCE and its easiness for interpreting. The four-factor solution (eigen values > 1) explained 34% of the variance. The factor loading matrix for this final solution is presented in Table 6. The factors identified were the following: 31 of the 32 items surrounding factors that affect SS loaded onto Factor 1, which was consequently labeled as *SS Factors*; 17 items surrounding Actual and Desired Individual SS, the only sexual factor contributing to SS (item 32 of the category: Sexual factors affecting SS) and some mainly individual sexual behaviors affecting SS (e.g., masturbation, using imagination, practicing a fantasy, using sex toys, erotic/pornographic literature and visual media), loaded onto Factor 2, thus labeled as *Individual SS*; 16 items surrounding Actual and Desired With-a-Partner SS, some non-individual sexual behaviors affecting SS (e.g., kissing, cuddling, hugging, giving/receiving oral sex, hetero-masturbation, vaginal coitus, petting, heavy-petting, pseudo-coitus), the two items considering sexual functioning and the item assessing sexuality importance loaded onto Factor 3, labeled as *With-a-Partner SS and Sexual Functioning*; and the four items assessing orgasm loaded onto Factor 4, which was labeled as *Orgasm*. All the items were retained since all of them contributed to at least a factor, and none of them failed to meet a primary factor loading of 0.2 or above. Items contributing to two or more factors were considered in the factor where the loadings were higher or, if lower, the differences were non-significative, except for Orgasm Frequency in Individual Practices, that contributed to *Orgasm* factor (0.20) and also had a high value in *Individual SS* factor (0.50).

Secondly, a CFA was conducted with ML method. As occurred with the EFA, the multifactoriality of the SSCI was demonstrated. Initially, five unobserved, exogenous variables (higher-order factors or constructs) were specified, each comprising the observed, endogenous variables listed in Table 2, Table 3 and Table 4 and the pre-established conceptual multidimensionality of the SSCI; namely, SS (comprising Actual With a Partner SS, Actual Individual SS, Desired With a Partner SS and Desired Individual SS), Orgasm Factors (comprising orgasm frequency with shared and non-shared activities, importance conceded to orgasm and overall satisfaction when orgasm), Sexual Functioning (comprising desire, excitement and importance conceded to a proactive, healthy and satisfactory sexuality), Sexual Factors (comprising the 32 items regarding factors related to sexuality and satisfaction) and Sexual Behaviors (comprising the 25 behaviors related to SS). We had to re-specify the model in order to increase its goodness of fit to the data. Inspired by the EFA results, satisfaction with sexual activities shared with a partner and satisfaction with non-shared, individual activities were separated in two dimensions. Similarly, sexual behaviors were also separated in behaviors in shared activities and individual activities. Moreover, the covariances between the high-order factors of Orgasm, Sexual Functioning, Sexual Factors and Sexual Behaviors with each other were non-significant, as well as the covariances between SS dimensions and Sexual Factors, and were removed from the model, leaving only the covariances of SS indicators with the remaining high-order factors, with the exception of the crossed covariances of SS dimensions and Sexual Behaviors.

**Figure 1:**
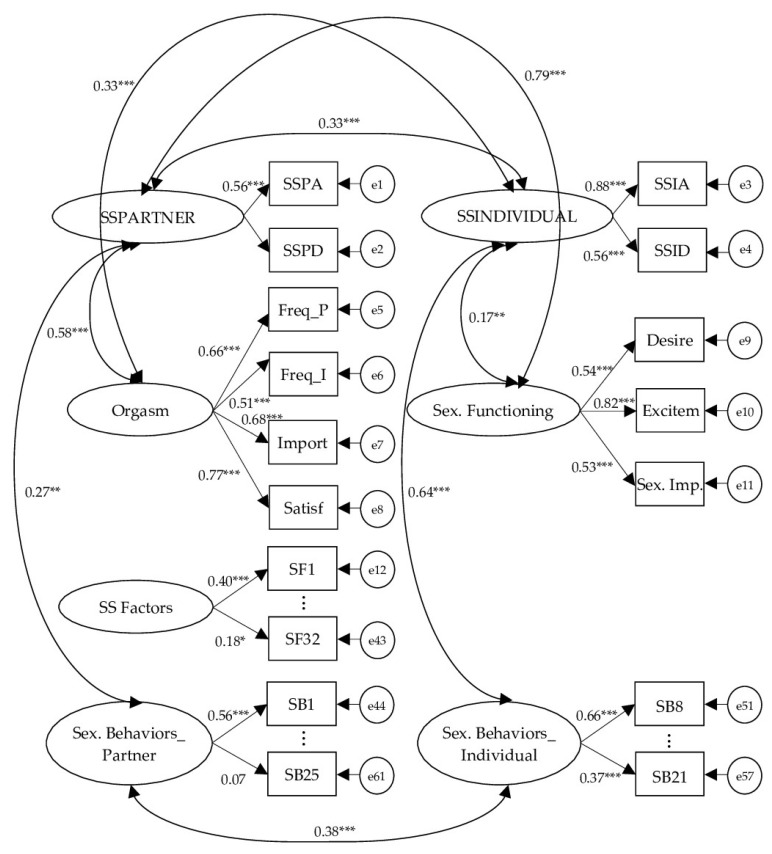
Path diagram of the factorial model from the CFA. (*** *p* < 0.001; ** *p* < 0.01; * *p* < 0.05.).

With such a recursive, overidentified model, Chi-square = 6316.121, *p* = 0.000, degrees of freedom = 2202, CMIN/df = 2.868 and RMSEA = 0.042, but CFI was <0.9. Based on the criteria for categorizing model fit, the obtained model exhibited “marginal” model-data fit. Figure 1 shows the standardized loadings; for the sake of easiness, the factor loadings for the Sexual Factors (all between 0.31 and 0.65, *p* < 0.001, excepting SF32 = 0.18, *p* < 0.05) and Sexual Behaviors observed variables (both with-a-partner and individual behaviors in their respective subdimension, all between 0.33 and 0.80, *p* < 0.001, excepting SB23 = 0.14 and SB24 = 0.16, *p* < 0.05; and SB25 = 0.07, *p* > 0.05) are omitted from the display.

Reliability for the global SSCI was appropriate, with Cronbach’s *alpha* = 0.89. Analyses also showed that the exclusion of none of the items would notably reduce or improve the internal consistency (*alpha*, if items were removed, ranged from 0.891 to 0.885). Reliability for each factor was also appropriate, showing Cronbach’s *alpha* = 0.81 for Factor 1: *SS Factors*; Cronbach’s *alpha* = 0.86 for Factor 2: *Individual SS*; Cronbach’s *alpha* = 0.83 for Factor 3: *With-a-Partner SS and Sexual Functioning and* Cronbach’s *alpha* = 0.73 for Factor 4: *Orgasm.*

## 4. Discussion

This study presented and explored the psychometric properties of the SSCI, an alternative multidimensional measure of SS that includes both the actual and desired levels of SS, considering not only the with-a-partner(s) but also the individual dimensions of SS, pursuing to go beyond the assessment of merely sexual functioning, partner-related issues and with-a-partner limited sexual behaviors, and aiming to understand varied and broader predictors of a healthy, fulfilling and pleasant sexuality. Thus, we explored its reliability and validity in a wide sample of young to mid-aged women, along with the main features of SS in this sample.

Cronbach’s alpha reliability coefficient for the SSCI was high, 0.89 for the global measure and internal consistencies were also appropriate for each of its factors. There is a general consensus in that values of 0.70 and above indicate an appropriate internal consistency. Some authors have proposed that values too near to 1 could involve redundant items that do not really add relevant information when assessing a construct [47]. Moreover, the reliability analysis revealed that this result would not be affected when any of the items was removed. These results indicate the SSCI is reliable and thus we could conclude that the information gathered with it is accurate.

We also performed other analyses to estimate SSCI psychometric characteristics. Firstly, we assessed the convergent validity of the SSCI with some of the most used measures to assess SS. As expected, ISS [10,33], NSSS-S [13,34] and FSFI [9,35] were intercorrelated, which is coherent given that all of them have proven to have appropriate psychometric characteristics and it is accepted that all of them measure SS, even when they are mostly focused on aspects such as intercourse, partner-related factors or respondent’s sexual functioning. All measures of SS of the SSCI were positively correlated with both ISS and NSSS-S. Additionally, the items regarding with-a-partner SS showed a relation with the selected three items regarding SS of the FSFI, which is coherent and was expected, given that those items only assess specifically with-a-partner SS. Our findings not only prove that our measure has a good convergent validity but also enhance the idea that SS variables of the SSCI measure the construct of SS going beyond the “with-a-partner” and the “intercourse” bias. In addition, a comparison of the descriptive results obtained with the SSCI satisfaction dimensions and the remaining tools of SS add value to the convergent validity of the SSCI.

Regarding the remaining variables of the SSCI, Orgasm Frequency With-a-Partner and Overall Satisfaction When Orgasm correlated with all ISS, NSSS-S and FSFI. This was expected, as those variables are intended to gather information on sexual functioning and ISS, NSSS-S and FSFI have some items assessing this construct (e.g., *“It is easy for me getting aroused with my partner”, “Our sexual life is very exciting”, “The quality of my orgasms”, “My body’s sexual functioning”, “My partner’s ability to orgasm”*). Moreover, the positive link between women’s frequency of orgasm and greater SS is highly consistent in the literature [48,49]. On the other hand, the absence of correlation of SSCI’s variable Orgasm Frequency in Individual Practices with FSFI is also logical and expected, since the FSFI refers to sexual experiences with a partner.

Unexpectedly, Orgasm Importance showed almost no significant relation with the external measures of SS, but did with almost all of the SSCI’s satisfaction dimensions. We define Orgasm Importance as the weight that reaching climax has in each woman’s experience of SS, but no other measure explores the value conceded to reaching climax, although some works have used measures aimed at studying the subjective orgasm experience, a complementary construct [50] and further study of its potential relations is advised. There is evidence that performance anxiety, concerns and other negative emotional states related with sexual functioning can negatively affect women’s ability to orgasm, so the more a woman focuses on the likelihood of reaching orgasm, the less likely she is to climax [51]. However, importance does not necessarily imply high, dysfunctional self-awareness, consciousness, concerns or interest in reaching climax when involving in a sexual behavior, which in turn could be dangerously related to anxiety or hypercontrol of the sexual response, thus decreasing SS. This is an interesting issue that should be fully investigated in the future. The relation of this item with NSSS-S and not the ISS or FSFI could be explained by the fact that the NSSS-S has some items assessing specifically ability or quality of orgasm whereas the other two do not.

Sexuality Importance correlated with all ISS, NSSS-S and FSFI, as well as with all the variables of the SSCI. This result was expected, since if a woman has a great interest in having a healthy, active and fulfilling sexuality, she would probably be more likely involved in enhancing her pleasure and satisfaction, maybe developing a greater frequency and variety of behaviors, both factors related with SS [30,52].

In addition, all the variables of the SSCI were intercorrelated, with some exceptions. The correlations found, as well as the lack of correlations, between the subdimensions of the SSCI, and ISS, NSSS-S and FSFI were completely expected, and support the idea that these variables assess completely different, yet complementary, aspects of females’ SS.

SSCI allows the researcher or clinician to describe in depth several aspects of women’s SS. Descriptive findings seem to indicate that the participants are a homogeneous group, with a quite satisfactory sexual life, both individually and, specially, with the partner(s). In fact, our findings reveal that this group of women not only consider that they obtain greater SS with their relationships with a partner, but also expect higher SS with the partner(s): even though these women would like to improve both levels, they are more interested in increasing their with-a-partner satisfaction than their individual SS. These results are congruent with those obtained in previous research with different samples [30]. It is also interesting to note that participants’ actual levels of satisfaction, both in individual and shared sexual experiences, are lower than those desired for themselves. This finding should make those responsible (institutions, clinicians, educators, researchers…) aware of the importance of spreading in the community interventions aimed at sexual education and sexual well-being promotion, particularly focused on young individuals and the female population, and surpassing the traditional “with-a-partner” and “intercourse” focus.

The rest of the SSCI dimensions allow to further explore these women’s interests regarding SS. The most satisfying behaviors were some of those which involved a partner, such as kissing, cuddling, hugging or vaginal intercourse. Furthermore, among the 32 factors related with SS assessed, those rated as more likely to affect these women’s satisfaction were those directly related with relationships (i.e., attraction to, and confidence with the sexual partner or the absence of a feeling of obligation to do something unwanted). These results are consistent with previous findings [21,53], since sexuality is mostly considered as a shared function that is undoubtedly related to confidence, emotion, attraction and other partner-related factors, and has commonly been studied within romantic relationships [54]. It is important to highlight that the feeling of obligation to have a sexual relationship or the belief that a woman has to accept or be receptive to the partner’s sexual approaches (sex-for-obligation, still a common motivation/drive for sex for women in heterosexual relationships) have been reported to predict lower SS [55], hence the importance of assessing these factors to collect data to plan educative or therapeutic interventions and research designs. It is also important to note that love or commitment factors are not among the features leading to increased SS, which supports that the participants can value the intimate factors of relationships in sexuality but do not necessarily equate them to romantic engagement [23].

Nevertheless, our results show that individual sexuality and its effects on SS also matter to this group of women: e.g., behaviors such as masturbation were rated as quite or very satisfactory, which tells us that these women often participate in and enjoy these behaviors. Concentration, experience and knowledge, self-esteem, body appreciation, being comfortable and contentment were some of the factors rated as having importance on sexuality, and they are some of the most interesting factors that can specially affect individual sexual behaviors or partnered-sexuality at an individual level. Individual sexual behaviors such as masturbation as well as its characteristics have become a frequent research topic in the last years ([56], also see [57] for a review) and results have been controversial (see [26] for a review). SSCI provides useful information on this topic, with items designed to assess different areas of individual sexuality. Complementary to these results, orgasm was always or almost always reached in individual sexual practices, more often than in with-a-partner practices, which reflects that most of the women who engage in this type of behaviors are usually able to reach climax. On the other hand, our results also show that, although it is still considered relevant, the majority of women do not consider orgasm as a conditio-sine-qua-non for achieving SS, and that those who do not experience it, still report a satisfactory sexual life.

Orgasm, being one of the human sexual response phases, has been considered an indicator of sexual functioning, one goal for sexual activities and an important source of SS [58,59]. Research on orgasm is rich and varied, although sometimes controversial: e.g., some results show that many women do not consider it central for their valuation of SS [60], while others have found that women who consider orgasm to be important are more likely to experience orgasms compared to those who think that it is not important to them [61]. These research findings indicate that, despite its significance, orgasm’s importance in women’s SS still needs to be addressed, hence the importance of measuring it, not only in terms of frequency, but also inquiring the overall value given to it, both in individual and shared sexual practices, and the impact that women perceive it has on their overall SS, when assessing SS. Moreover, as our findings also seem to indicate that the most satisfying—shared or non-shared—activities are those with the highest frequency (as well as those less satisfactory are those less practiced), future research is necessary to explore whether people engage in those behaviors which are the most pleasurable for them, or rate as most pleasurable those activities in which they are involved, an issue that has not been fully addressed yet [26].

Complementary to the above-mentioned findings, it is worthy to note that the women participating in the present study reported appropriate sexual functioning in terms of orgasm but also in terms of desire and excitement. More importantly, the importance conceded to having a healthy, agentic, fulfilling and satisfactory sexuality is very high for the majority of the participants.

Finally, we conducted both an EFA and a CFA to explore the structure of the SSCI, both supporting its multidimensionality. In the EFA, four factors surrounding *Individual SS*, comprising also mainly individual sexual behaviors, *With-a-Partner SS and Sexual Functioning*, comprising also mainly shared sexual behaviors and sexual functioning factors, *Factors affecting SS* and *Orgasm factors* were obtained which explained 34% of the data variance. While the indicators’ weights were appropriate in their respective dimension for the vast majority of cases, the EFA also showed some items unexpectedly aligned into factors different to those anticipated, namely some non-individual sexual behaviors (e.g., pinching or using ties, domination, threesomes or dogging) contributing to the individual SS factor instead of the with-a-partner SS one. This could be due to the fact that these behaviors are the least performed ones and the least contributing to any type of SS—some of these behaviors are not performed by 91% of the sample—and these low scores might be affecting the loadings of these concrete items on the EFA, causing this particular grouping. The same explanation could be given to other items referring to shared activities that loaded onto individual SS such as “giving or receiving oral anal sex”, which was also reported as one of the less frequent behaviors (63%), but in this particular case, the formulation of the item might also be contributing to its grouping with individual behaviors affecting SS, as asking how “giving” or “receiving” oral anal sex affects SS might focus the attention on the individual, subjective aspects of the behavior (the pleasure experienced by the person during the behavior), disregarding the partner contribution or obliterating that a partner is needed to perform this behavior. Moreover, the Sexual Factor 32, “How much does using fantasies, erotic toys, etc. influence your sexual satisfaction?”, contributed to the factor individual SS instead of the SS factors one. The dimension *Factors affecting SS* comprises a heterogeneous cluster of cognitive, emotional, motivational, behavioral, individual, partner-related topics (e.g., emotions, feelings, experiences, abilities and personal circumstances) with the only common aspect being their possibility to affect SS. The alignment of this item with *Individual SS* factor could be due to a couple of possibilities: firstly, this item could be easily misperceived as an individual behavior affecting SS, as fantasies and erotic toys are often used during individual sexual behaviors, such as masturbation, e.g., some research shows that women use sex toys significantly more often in solo sex than men [62]; secondly, fantasies are undoubtedly a personal, subjective, therefore, individual cognition that can affect SS. To solve these problematic behaviors, these items could be reformulated; however, all these items were afterwards correctly identified by the CFA in their respective dimensions.

All these items regarding sexual factors and behaviors were included in the SSCI in order to assess a wide range of sexual activities and factors (individual and with-a partner ones) from the most common ones (e.g., kissing) to the least common ones (e.g., swinging). This decision had two main aims: first, to be able to describe in depth the sexuality of all the people/everyone answering the measure, inquiring not just about the frequency of a certain sexual behavior, but the impact of each one on people’s SS, and secondly, to use these data to better understand the relationships between these factors and behaviors, their predictability and their explanatory potential on SS. This is meant to include the nowadays rapidly changing, fluid, creative and multifaceted sexuality-related tendencies. Hence our interest in maintaining all the items although their factor loadings were unusual, as future research is aimed at focusing on a broader range of populations (e.g., clinical, non-clinical, men, older people, LGTBIQ+ populations and non-monogamous relationships). Nonetheless, rewriting some of these items is advisable to overcome some of the above-mentioned difficulties, e.g., creating two different items, one for “giving” and other for “receiving” activities or separating the individual dimension of the experiences from the shared dimension. Additionally, SSCI’s item about level of satisfaction produced with different individual and partnered sexual behaviors could be used in combination with an item assessing the frequency of performance of these same behaviors, thus obtaining a measure of behavior and a measure of SS, adding interesting data to both research and clinical practice.

The item assessing Orgasm Frequency with Individual Practices contributed more to the *Individual SS* factor than to *Orgasm* factor, and the items describing sexual function aligned in the *With-a-Partner SS* instead of grouping around a sexual functioning factor, although these items’ formulation asked specifically about desire and excitement both in shared or individual sexual practices. It has been proposed [63] that two items with similar loadings in the same factor could be inter-related given an existing relationship between them (e.g., between individual orgasm frequency and individual SS), and not necessarily because they assess the same construct; so, it is possible that the first mentioned alignment responds to this circumstance. On the other hand, sexual functioning has been largely conceptualized as the ability to perform successfully in a sexual activity, and has been concentrated mainly on sexual dysfunctions (such as capability for sexual intercourse or sexual interest) affecting mainly sexual behaviors with the partner [64], so the alignment of these items within the partnered SS factor could also respond to this issue.

Alternatively, although the sample is quite heterogeneous in their characteristics, this particular group of women’s SS is quite homogeneous, which can affect EFA analyses, since in this type of analysis, more heterogeneity of the sample makes it easier to show the correlation between items and EFA’s results interpretation [63]. However, each of the factors could probably be strengthened through revision (rewriting) of items with lower primary loadings and possibly adding new items, e.g., reformulating the item “How much does whatever you do (the techniques you use) influence your sexual satisfaction…?” into a more particular version, such as “How much do the sexual behaviors you put into practice influence your sexual satisfaction?”; or instead of “using fantasies, sex toys, etc.”, state: “fantasizing, using sex toys individually or with your partner”.

The CFA confirmed a multidimensional structure, with the latent constructs assessed by the SSCI corresponding to Individual SS, With-a-Partner SS, Sexual Functioning, Orgasm Factors, SS Factors and Sexual Behaviors, sub-grouped into individual and with-a-partner activities, and these factors were internally consistent. The path analysis showed the covariances between SS dimensions and all high-order factors except SS factors. Since the selection of these particular items for the SS factors, which can be viewed as heterogeneous but highly intercorrelated indicators measuring different experiences, emotions, abilities, expectations and other cognitive and behavioral features, was made following previous qualitative research [30] in which women were asked to inform about topics they thought affect their SS, and as the only feature in common between all of them is that they inquire about the impact on SS, the unexpected lack of covariance between this factor and SS dimensions must be confirmed in future research. However, this dimension explores a variety of factors with a possible impact on SS within a comprehensive assessment of sexual pleasure and health, and could be used or not depending on the practitioner’s or researcher’s interests. On the other hand, high-order factors pertaining orgasm, sexual functioning, sexual factors and sexual behaviors did not covariate with each other, although all of them were correlated with Individual and With-a-Partner SS. This finding was moderately expected due to SSCI assessing different aspects of sexuality with a focus on SS.

Analyses confirmed the multidimensionality of the SSCI, a tool evaluating aspects very different from each other such as satisfaction with sexual activities of different kinds including both solitary and shared ones and actual and desired pleasure, orgasm factors, sexual functioning factors, relationship, activity and personal factors that affect satisfaction and sexual behaviors that can be more or less satisfying. Particularly, the dimensions of sexual factors and sexual behaviors affecting SS can be viewed as complementary dimensions to understand SS, which can offer interesting information on the person’s sexuality, and serve as indicators of predictors of SS. In turn, orgasm factors and sexual functioning factors are particularly relevant for SS when both shared and solitary activities are conducted. Finally, our findings stress the relative independence of satisfaction when shared and solitary activities are conducted, indicating that both dimensions of SS should be observed when satisfaction with sexuality is to be assessed, explored or intervened. Considered together, our findings support the use of the SSCI as a multifaceted, comprehensive tool to gather information on SS and the varied contributing or related experiences of sexual pleasure.

Nonetheless, some limitations must be noted. Our findings were obtained with a sample of young and mid-aged females and replication with a more heterogeneous sample is advisable. Future complementary lines of action could focus on its application to other samples (men, LGTBI+, different cultural and age groups, etc.) in order to support SSCI’s usefulness in multi-identity and multi-sexuality scenarios. Moreover, self-report-based studies such as the present one might be completed with other assessment procedures and sources (e.g., psychophysiological measures, partners’ reports). In addition, this is a cross-sectional study with validation purposes, and future research (e.g., longitudinal research to explore life-course variations, experimental research to study enhancements in SS after an intervention) is needed to further support the utility and soundness of the SSCI in varied contexts and with different purposes.

## 5. Conclusions

We have developed and validated a multidimensional SS measure aiming to assess SS going beyond the old-fashioned conceptualization of SS as being a simple consequence of the absence of a sexual dysfunction, the need of the presence of a partner to achieve SS, the focus on intercourse and the bias of heteronormativity, and including the importance given to a satisfactory, agentic and healthy sexuality and the desired experiences of satisfaction related to sex and sexuality. The SSCI is a useful instrument with appropriate psychometric properties, that can be used both in educative, clinical and research contexts, being able to draw a comprehensive description of people’s sexuality, including the pleasure obtained from both individual and shared sexual experiences, the desire to improve their SS, the importance given to certain factors affecting SS, the impact of a broad variety of sexual behaviors on SS and—but not solely—factors related with sexual functioning and orgasm. Based on all the results, we feel that the researcher or the clinician can find in the SSCI a measure that can help in assessing several dimensions of sexuality and satisfaction, and that can be used as a whole form or in any of its parts depending on the purposes of the assessment process.

## Figures and Tables

**Table 1:** Socio-demographic data and participants’ relationship/sexual experiences (N = 1080).

Educational Level	N	%
Uneducated	2	0.2
Primary School	12	1.1
Secondary School	23	2.1
Professional Training	53	4.9
University	862	79.8
University Postgraduate (Master/PhD)	128	11.9
**Religion/Spiritual Factors**	**N**	**%**
Catholic	477	44.1
Buddhist	7	0.6
Muslim	6	0.6
Protestant	5	0.5
Agnostic	149	13.8
Atheist	433	40.1
Other	3	0.3
**Level of Religiosity**	**N**	**%**
Not religious at all	532	49.3
Not very/A little religious	381	35.3
Quite religious	121	11.2
Very religious	46	4.2
**Political Ideology**	**N**	**%**
None	128	11.9
Conservative	33	3.1
Centre	198	18.3
Progressive	533	49.4
Not identified by the participant	188	17.3
**Cohabitation**	**N**	**%**
Alone	56	5.2
With friends	375	34.7
With partner	143	14.8
With family of origin (parents, siblings…)	444	39.6
With family of procreation (offspring)	57	5.2
Student residence	5	0.5
**Number of Sporadic Sexual Partners**	**N**	**%**
None	487	45.1
1 to 5	315	29.2
5 to 10	144	13.3
10 to 20	104	9.6
More than 20	30	2.8
**Number of Committed Sexual Partners**	**N**	**%**
None	368	34.1
1 to 5	621	57.5
5 to 10	73	6.7
10 to 20	16	1.5
More than 20	2	0.2

**Table 2:** Descriptive results of all study variables.

Variable (Possible Scores Range)	M ± SD [Min–Max in the Sample]
Actual Individual Sexual Satisfaction (0–3)	2.09 ± 0.83 [0–3]
Actual With the Partner(s) Sexual Satisfaction (0–3)	2.23 ± 0.70 [0–3]
Desired Individual Sexual Satisfaction (0–3)	2.6 ± 0.83 [0–3]
Desired With the Partner(s) Sexual Satisfaction (0–3)	2.9 ± 0.33 [0–3]
Orgasm Frequency With a Partner(s) (0–3)	2.05 ± 0.97 [0–3]
Orgasm Frequency in Individual Practices (0–3)	2.17 ± 1.11 [0–3]
Orgasm Importance (0–4)	2.31 ± 0.77 [0–4]
Overall Satisfaction When Orgasm (0–5)	3.14 ± 1.25 [0–5]
Desire Level (0–4)	2.95 ± 0.77 [0–4]
Excitement Level (0–4)	3.01 ± 0.74 [0–4]
Sexuality Importance (0–3)	2.18 ± 0.71 [0–3]
Factors that affect Sexual Satisfaction ^a^ (0–3)	2.12 ± 0.36 [1–3]
Sexual Behaviors that affect Sexual Satisfaction ^a^ (0–4)	1.34 ± 1.2 [0–4]
ISS ^a^ (1–7)	5.68 ± 0.93 [1–7]
NSSS-S ^a^ (1–5)	3.81 ± 0.70 [1–5]
FSFI ^a^ (0–5)	3.95 ± 1.18 [1–5]

^a^ Average.

**Table 3:** Descriptive results and frequencies for the dimension Factors that affect Sexual Satisfaction.

How Much Do the Following Aspects Influence Your Sexual Satisfaction…?]	It Does Not Affect Me at All (%)	It Only Affects Me a Little (%)	It Affects Me Somewhat (%)	It Affects Me a Lot (%)	M ± SD [Min–Max in the Sample]
...whatever I do (e.g., the techniques I use)	3.5	18.3	50.7	27.5	2.02 ± 0.77 [0–3]
...whatever others do when they are with me (e.g., the techniques my sexual partner(s) use)	0.4	5.2	40.6	53.7	2.48 ± 0.62 [0–3]
…communication (i.e., stating what I like/dislike or I want/don’t want to do)	0.9	14.4	45.4	39.3	2.23 ± 0.72 [0–3]
...feeling attracted to my sexual partner(s)	0	2.6	24.9	72.5	2.70 ± 0.51 [1–3]
...desiring to have a sexual relationship, sexual activity	0.4	5.2	30.6	63.8	2.58 ± 0.61 [0–3]
…my concentration levels, not dwelling on something else	2.6	20.1	31	46.3	2.21 ± 0.85 [0–3]
…the experience/knowledge I/we have	5.2	26.2	45.4	23.1	1.86 ± 0.83 [0–3]
...my own ability	4.4	27.9	47.2	20.5	1.84 ± 0.80 [0–3]
…my sexual partner’s (partners’) ability	2.2	15.3	48.5	34.1	2.14 ± 0.75 [0–3]
…my disposition to try new things (e.g., innovation, creativity)	2.2	21.8	46.7	29.3	2.03 ± 0.77 [0–3]
…feeling relaxed/comfortable, unbothered by anything/anyone	1.3	9.2	38	51.5	2.40 ± 0.71 [0–3]
…the ability to express/say pretty/pleasant things to my sexual partner(s) or my sexual partner’s (partners’) ability to do that to me	6.1	27.5	34.1	32.3	1.93 ± 0.92 [0–3]
…the ability to express/say erotic things to my sexual partner(s) or my sexual partner’s (partners’) ability to do that to me	5.7	17.5	36.7	40.2	2.11 ± 0.89 [0–3]
…experiencing sexuality as something normal/healthy/desirable	1.7	5.2	27.5	65.5	2.57 ± 0.58 [0–3]
…not feeling obligated to do something I don’t want/desire to do	2.6	4.4	21	72.1	2.62 ± 0.69 [0–3]
…making something/someone horny	3.5	16.6	41.9	38	2.14 ± 0.82 [0–3]
…having confidence with my sexual partner(s)	1.3	5.2	26.6	66.8	2.6 ± 0.65 [0–3]
…the contraceptive method used	21.4	38.4	26.2	14	1.33 ± 0.96 [0–3]
…loving my sexual partner(s) or being loved by her/him/them	9.2	26.2	25.8	38.9	1.94 ± 1 [0–3]
…the faith/truth/security with my sexual partner(s)	0	7.4	32.8	59.8	2.52 ± 0.63 [1–3]
…my self-esteem levels	1.7	7.9	36.2	54.1	2.43 ± 0.71 [0–3]
…feeling comfortable with my body/not feeling ashamed	0.9	7.9	27.9	63.3	2.54 ± 0.58 [0–3]
…me reaching climax	6.6	29.7	44.1	19.7	1.77 ± 0.84 [0–3]
…me and my partner(s) reaching climax	4.4	20.5	45	30.1	2.01 ± 0.83 [0–3]
…the place where the encounter takes place (e.g., noise, peace, the chance of getting caught deliberately or not, interruptions)	4.4	23.1	36.7	35.8	2.04 ± 0.87 [0–3]
…being very sexually excited	0.9	7	36.2	55.9	2.74 ± 0.67 [0–3]
…feeling that my sexual partner(s) is committed to the relationship	26.6	38.9	20.5	14	1.22 ± 0.99 [0–3]
…feeling contented or happy	1.3	12.7	40.2	45.9	2.31 ± 0.74 [0–3]
…me and my partner(s) having the same level of desire	11.8	36.7	35.4	16.2	1.56 ± 0.90 [0–3]
…having respect for, faithfulness to each other	3.1	12.2	35.4	49.3	2.31 ± 0.80 [0–3]
…not being tired	3.9	25.8	38.4	31.9	1.98 ± 0.86 [0–3]
…using sexual fantasies, role-playing, erotic toys...	22.7	49.3	19.7	8.3	1.14 ± 0.86 [0–3]

**Table 4:** Descriptive results and frequencies for the dimension Sexual Behaviors that affect Sexual Satisfaction.

How Much Do the Following Behaviors Influence Your Sexual Satisfaction…?	I Do Not Practice It (%)	Not Satisfactory at All (%)	Somewhat Satisfactory (%)	Quite Satisfactory (%)	Very Satisfactory (%)	M ± SD [Min–Max in the Sample]
Kissing	0.4	0.4	1.3	20.2	77.6	3.74 ± 0.55 [0–4]
Cuddling	0	0.4	1.3	19.7	78.5	3.76 ± 0.48 [1–4]
Hugging	0	0.9	6.6	20.6	71.9	3.64 ± 0.65 [1–4]
Licking or biting my sexual partner(s), being licked/bitten by her/him/them	0.4	0	8.3	28.5	62.7	3.53 ± 0.58 [0–4]
Giving oral sex (i.e., fellatio/cunnilingus)	3.5	6.1	14	36.8	39.5	3.03 ± 1.05 [0–4]
Receiving oral sex (i.e., cunnilingus)	5.3	3.1	10.5	21.9	59.2	3.27 ± 1.11 [0–4]
Giving or receiving anal oral sex (i.e., anilingus)	63.6	6.1	13.6	7	9.6	.93 ± 1.39 [0–4]
Masturbation (i.e., manual self-stimulation of the own genitalia)	7.9	3.9	14.9	34.2	39	2.93 ± 1.19 [0–4]
Hetero-masturbation (i.e., manual stimulation of sexual partner’s(partners’) genitalia, or being manually stimulated on the genitalia by the partner(s), simultaneously or not)	1.8	13	12.7	39	45.2	3.25 ± 0.86 [0–4]
Vaginal intercourse (i.e., penis-vagina introduction)	3.9	1.3	5.3	18.9	70.6	3.51 ± 0.95 [0–4]
Anal intercourse (i.e., penis-anus introduction)	62.3	10.5	14.5	9.2	3.5	0.81 ± 1.2 [0–4]
Using my imagination/fantasies regarding other contexts, situations, people…	10.5	14.5	35.1	25	14.9	2.19 ± 1.17 [0–4]
Putting into practice any sexual fantasy, practicing it	13.2	7.9	18.4	36.8	23.7	2.5 ± 1.3 [0–4]
Erotic play, seducing my partner(s) (e.g., flirting, performing a striptease)	9.2	5.3	24.6	35.5	25.4	2.63 ± 1.19 [0–4]
Petting	1.8	1.8	19.3	40.4	36.8	3.09 ± 0.87 [0–4
Heavy petting	1.8	.9	8.3	42.1	46.9	3.32 ± 0.8 [0–4]
Pseudo-coitus (i.e., imitate intercourse movements)	6.6	8.8	27.2	32.9	24.6	2.60 ± 1.14 [0–4]
Using sex toys (e.g., bullet vibrator, “satisfier”, penis rings, clitoral stimulators)	36.8	7.5	21.5	18.9	15.4	1.68 ± 1.05 [0–4]
Using erotic/pornographic literature (e.g., novels, comics)	42.5	11.4	26.8	12.3	7	1.30 ± 1.32 [0–4]
Using erotic/pornographic visual media (e.g., movies, photos, videos)	31.6	12.3	26.8	17.5	11.8	1.66 ± 1.39 [0–4]
Engaging in sexual practices aimed to delay or impede climax (e.g., tantric sex)	66.2	6.6	11	9.2	7	0.84 ± 1.32 [0–4]
Engaging in more provocative but non-risky sexual practices (e.g., pinching, using ties, slapping, covering the eyes)	26.8	6.1	18	27.2	21.9	2.11 ± 1.5 [0–4]
Engaging in more aggressive sexual practices, ones that require safe-words (e.g., BDSM)	82.9	3.5	6.1	4.4	3.1	0.41 ± 1 [0–4]
Engaging in sex with more than one sexual partner (e.g., threesomes, swinging, orgies, dogging)	82	4.8	3.5	4.8	4.8	0.46 ± 1.09 [0–4]
Engaging in potentially risky sexual practices (e.g., with unknown people, in dark rooms, sex roulettes)	90.8	3.5	2.6	2.2	0.9	0.19 ± 0.67 [0–4]

**Table 5:** Pearson’s zero-order correlations for the study variables.

Variable	2	3	4	5	6	7	8	9	10	11	12	13	14	15	16
1. Actual Individual SS	0.186 **	0.502 **		0.162 **	0.638 **	0.146 **	0.231 **		0.184 **	0.131 **			0.134 **	0.228 **	
2. Actual With the Partner(s) SS	-	0.074 *	0.185 **	0.566 **	0.160 **	0.146 **	0.238 **	0.264 **	0.468 **	0.265 **			0.576 **	0.644 **	0.529 **
3. Desired Individual SS		-	0.231 **		0.399 **		0.099 **			0.102 **	0.204 **		0.072 *	0.092 *	
4. Desired With the Partner(s) SS			-	0.119 **	0.092 **	0.096 **	0.113 **	0.169 *	0.171 **	0.241 **			0.121 **	0.120 **	0.151 **
5. Orgasm Freq. With a Partner(s)				-	0.366 **	0.409 **	0.481 **		0.220 **	0.210 **			0.365 **	0.533 **	0.326 **
6. Orgasm Freq. in Individual Practices					-	0.275 **	0.367 **	0.135 *	0.136 *	0.142 **			0.130 **	0.235 **	
7. Orgasm Importance						-	0.606 **			0.098 **				0.174 **	
8. Overall Satisfaction When Orgasm							-	0.136 *	0.185 **	0.192 **	0.173 **		0.127 **	0.267 **	0.091 *
9. Desire Level								-	0.420 **	0.309 **					
10. Excitement Level									-	0.380 **	0.142 *				
11. Sexuality Importance										-	0.254 **		0.272 **	0.298 **	0.197 **
12. Factors that affect SS ^a^											-				
13. Sexual Behaviors that affect SS ^a^												-			
14. ISS ^a^													-	0.777 **	0.607 **
15. NSSS-S ^a^														-	0.564 **
16. FSFI ^a^															-

Note. ^a^ Average. SS Sexual Satisfaction. Values displayed are significant at ** *p* < 0.01 and * *p* < 0.05.

**Table 6:** Orthogonally rotated factor loadings for the SSCI items in the EFA.

	Factor 1 *SS Factors*	Factor 2 *Individual SS*	Factor 3 *With-a-Partner SS and Sexual Functioning*	Factor 4 *Orgasm*
Actual Individual Sexual Satisfaction		0.475		
Actual With the Partner(s) Sexual Satisfaction			0.450	
Desired Individual Sexual Satisfaction		0.454		
Desired With the Partner(s) Sexual Satisfaction			0.249	
Orgasm Frequency With a Partner(s)				0.682
Orgasm Frequency in Individual Practices				0.202
Orgasm Importance				0.523
Overall Satisfaction When Orgasm				0.634
Desire Level			0.323	
Excitement Level			0.496	
Sexuality Importance			0.395	
Factors that affect SS 1	0.298			
Factors that affect SS 2	0.406			
Factors that affect SS 3	0.323			
Factors that affect SS 4	0.352			
Factors that affect SS 5	0.457			
Factors that affect SS 6	0.471			
Factors that affect SS 7	0.419			
Factors that affect SS 8	0.361			
Factors that affect SS 9	0.433			
Factors that affect SS 10	0.428			
Factors that affect SS 11	0.493			
Factors that affect SS 12	0.521			
Factors that affect SS 13	0.528			
Factors that affect SS 14	0.369			
Factors that affect SS 15	0.377			
Factors that affect SS 16	0.350			
Factors that affect SS 17	0.444			
Factors that affect SS 18	0.342			
Factors that affect SS 19	0.425			
Factors that affect SS 20	0.512			
Factors that affect SS 21	0.493			
Factors that affect SS 22	0.558			
Factors that affect SS 23	0.414			
Factors that affect SS 24	0.429			
Factors that affect SS 25	0.426			
Factors that affect SS 26	0.462			
Factors that affect SS 27	0.465			
Factors that affect SS 28	0.632			
Factors that affect SS 29	0.377			
Factors that affect SS 30	0.484			
Factors that affect SS 31	0.323			
Factors that affect SS 32		0.427		
Sexual Behaviors that affect SS 1 (P)			0.728	
Sexual Behaviors that affect SS 2 (P)			0.760	
Sexual Behaviors that affect SS 3 (P)			0.548	
Sexual Behaviors that affect SS 4 (P)			0.554	
Sexual Behaviors that affect SS 5 (P)			0.338	
Sexual Behaviors that affect SS 6 (P)			0.403	
Sexual Behaviors that affect SS 7 (P)		0.502		
Sexual Behaviors that affect SS 8 (I)		0.517		
Sexual Behaviors that affect SS 9 (P)			0.540	
Sexual Behaviors that affect SS 10 (P)			0.424	
Sexual Behaviors that affect SS 11 (P)		0.488		
Sexual Behaviors that affect SS 12 (I)		0.574		
Sexual Behaviors that affect SS 13 (I)		0.557		
Sexual Behaviors that affect SS 14 (P)		0.416		
Sexual Behaviors that affect SS 15 (P)			0.470	
Sexual Behaviors that affect SS 16 (P)			0.616	
Sexual Behaviors that affect SS 17 (P)			0.338	
Sexual Behaviors that affect SS 18 (I)		0.590		
Sexual Behaviors that affect SS 19 (I)		0.542		
Sexual Behaviors that affect SS 20 (I)		0.605		
Sexual Behaviors that affect SS 21 (P)		0.495		
Sexual Behaviors that affect SS 22 (P)		0.465		
Sexual Behaviors that affect SS 23 (P)		0.522		
Sexual Behaviors that affect SS 24 (P)		0.575		
Sexual Behaviors that affect SS 25 (P)		0.383		
Eigen value	9.656	6.588	3.818	3.084
% Explained variance	14.200	9.688	5.614	4.535
% Explained cumulative variance	34.038			

Extraction method: Maximum Likelihood. Rotation method: Varimax with Kaiser normalization. Rotation has converged in 7 iterations. Cutting loading weights: >0.20. SS: Sexual Satisfaction; P: Behavior with a partner; I: Individual behavior.

## Appendix A

INDICADOR INTEGRAL DE SATISFACCIÓN SEXUAL (IISS)
[SEXUAL SATISFACTION COMPRENHENSIVE INDEX (SSCI)]

La satisfacción sexual es el grado de placer, bienestar y plenitud que experimenta una persona en relación con su actividad sexual. A continuación, realizaremos una serie de preguntas sobre tu satisfacción sexual. En cada caso, te pedimos que indiques tu opinión y experiencias respecto a la pregunta planteada, es decir, lo que sea más aplicable a tu caso. [Sexual satisfaction is the degree of pleasure, well-being and fullness that a person experiences in relation to their sexual activity. Below, we will ask you a series of questions about your sexual satisfaction. In each case, we ask you to indicate your opinion and experiences regarding the question posed, namely the option more applicable to you].

Aclaración: En las siguientes preguntas, “pareja sexual” se refiere a cualquier otra persona con la que realizas/has realizado actividades sexuales de la naturaleza que sea, en el contexto de una relación romántica o no. Además, como podemos tener sexo con más de una persona a la vez, lo indicamos con plural [Note: In the following questions, “sexual partner” refers to any other person with whom you engage/have engaged in sexual activities of any nature, in the context of a romantic relationship or not. Moreover, since we can have sex with more than one person at a time, this is noted with the plural]

1. ¿Podrías decirnos cómo de satisfactorias son en general tus relaciones sexuales con otra(s) persona(s), o en pareja? (Si no tienes pareja(s) sexual(es) en la actualidad, piensa en tus anteriores relaciones en general) [Could you tell us how satisfactory your sexual relationships with a partner(s) are in general? (If you don´t have any sexual partner(s) currently, think about your past relationships in general)]

☐ Nada satisfactorias/No me interesa [Not satisfactory at all/Not interested in it]
☐ Poco satisfactorias [Quite unsatisfactory]
☐ Bastante satisfactorias [Quite satisfactory]
☐ Muy satisfactorias [Very satisfactory]

2. ¿Podrías decirnos cómo de satisfactorias son en general tus prácticas sexuales individuales? (Como la masturbación, etc.) [Could you tell us how satisfactory your individual sexual practices are in general? (Such as masturbation, etc.)]

☐ Nada satisfactorias/No me interesa [Not satisfactory at all/Not interested in it]
☐ Poco satisfactorias [Quite unsatisfactory]
☐ Bastante satisfactorias [Quite satisfactory]
☐ Muy satisfactorias [Very satisfactory]

3. ¿Podrías decirnos cómo de satisfactorias desearías que fueran tus relaciones sexuales con otra(s) persona(s), o en pareja? [Could you tell us how satisfying would you like your sexual relationships with a partner(s) to be?]

☐ Nada satisfactorias/No me interesa [Not satisfactory at all/Not interested in it]
☐ Poco satisfactorias [Quite unsatisfactory]
☐ Bastante satisfactorias [Quite satisfactory]
☐ Muy satisfactorias [Very satisfactory]

4. ¿Podrías decirnos cómo de satisfactorias desearías que fueran tus prácticas sexuales individuales? (Como la masturbación, etc.) [Could you tell us how satisfying would you like your individual sexual practices to be? (Such as masturbation, etc.)]

☐ Nada satisfactorias/No me interesa [Not satisfactory at all/Not interested in it]
☐ Poco satisfactorias [Quite unsatisfactory]
☐ Bastante satisfactorias [Quite satisfactory]
☐ Muy satisfactorias [Very satisfactory]

5. ¿Con qué frecuencia alcanzas el orgasmo durante tus prácticas sexuales con otra(s) persona(s), o en pareja? (Como coito, sexo oral, etc.). Piensa en el momento actual, y si no tienes pareja sexual, en tus prácticas pasadas [How often do you reach orgasm during your sexual practices with a partner(s)? (e.g., intercourse, oral sex, etc.). Think about the present, and if you do not have any sexual partner(s), think of your past experiences]

☐ Nunca he tenido un orgasmo [I have never reached an orgasm]
☐ A veces [Sometimes]
☐ Bastantes veces [Quite a few times]
☐ Casi siempre o siempre [Almost always or always]

6. ¿Con qué frecuencia alcanzas el orgasmo durante las prácticas sexuales individuales (como la masturbación, etc.)? [How often do you reach orgasm during your individual sexual practices (e.g., masturbation, etc.)?]

☐ Nunca he tenido un orgasmo [I have never reached an orgasm]
☐ A veces [Sometimes]
☐ Bastantes veces [Quite a few times]
☐ Casi siempre o siempre [Almost always or always]

7. Alcanzar el orgasmo ¿hace que tus prácticas sexuales, compartidas o individuales, sean más satisfactorias? [Does reaching orgasm make your sexual practices, shared or individual ones, more satisfying?]

☐ No. Disfruto plenamente con mis prácticas sexuales sin orgasmos [No. I fully enjoy my practices without having an orgasm]
☐ No. Realmente me da igual si tengo orgasmos o no, lo que me hace quedar satisfecha son otros factores [No. I really don’t care if I have orgasms or not, what makes me satisfied are other factors]
☐ No necesariamente. Me gusta más cuando tengo orgasmos, pero puedo disfrutar una práctica que no termina en orgasmo y sentirme satisfecha al terminar [Not necessarily. I like it better when I have orgasms, but I can enjoy a practice that does not end in orgasm and feel satisfied with it]
☐ Sí, generalmente sólo me siento completamente satisfecha si logro tener al menos un orgasmo [Yes, I usually only feel fully satisfied if I manage to have at least one orgasm]
☐ Sí, generalmente sólo me siento completamente satisfecha si logro tener más de un orgasmo [Yes, I usually only feel fully satisfied if I manage to have more than one orgasm]

8. ¿Cómo de satisfactorias son tus prácticas sexuales, compartidas o individuales, cuando alcanzas al menos un orgasmo? [How satisfactory are your sexual practices, shared or individual ones, when you reach at least one orgasm?]

☐ Generalmente no tengo orgasmos, así que disfruto con mis prácticas sin orgasmos [I usually don’t have orgasms, so I enjoy my practices without orgasms]
☐ Igual que cuando no lo alcanzo, mi satisfacción no depende en absoluto de tener orgasmos [Just like when I don’t reach it, my satisfaction doesn’t depend on having orgasms at all]
☐ Algo más satisfactorias [Somewhat more satisfactory]
☐ Bastante más satisfactorias [Quite more satisfactory]
☐ Mucho más satisfactorias [Much more satisfactory]
☐ Yo sólo me siento completamente satisfecha si logro tener al menos un orgasmo [I only feel completely satisfied if I manage to have at least one orgasm]

9. En general, tu nivel de deseo a la hora de realizar comportamientos sexuales, individuales o con pareja(s), es... [In general, your level of desire to perform sexual practices, shared or individual ones, is...]

☐ Nada de deseo [Null]
☐ Muy bajo [Very low]
☐ Moderado [Moderate]
☐ Alto [High]
☐ Muy alto [Very high]

10. En general, tu nivel de excitación a la hora de realizar comportamientos sexuales, individuales o con pareja(s), es... [In general, your level of sexual arousal to perform sexual practices, shared or individual ones, is...]

☐ Nada de excitación [Null]
☐ Muy bajo [Very low]
☐ Moderado [Moderate]
☐ Alto [High]
☐ Muy alto [Very high]

11. A continuación te mostramos una lista con aspectos que la gente piensa que pueden afectar a su satisfacción sexual. ¿Podrías indicarnos en qué medida crees que cada uno de estos factores afecta a tus niveles de satisfacción sexual? (En otras palabras, ¿depende, en tu caso, tu satisfacción sexual de estos aspectos? [We will now show you a list of features that people usually think can affect their sexual satisfaction. Could you tell us to what extent do you think each of these factors affect your levels of sexual satisfaction? (In other words, in your case, would your sexual satisfaction depend on the following features?)])

¿En qué medida lo siguiente influye en tu satisfacción sexual...? [How much do the following aspects influence your sexual satisfaction…?]	No me afecta en nada [It does not affect me at all]	Me afecta algo [It only affects me a little]	Me afecta bastante [It affects me somewhat]	Me afecta mucho [It affects me a lot]
…lo que hagas tú (p.e., las técnicas que uses) [...whatever I do (e.g., the techniques I use)]				
…lo que hagan otras personas cuando están conmigo (p.e., las técnicas que usan) [...whatever others do when they are with me (e.g., the techniques my sexual partner(s) use)]				
…la comunicación (esto es, decir/transmitir lo que te gusta/no te gusta o lo que te apetece/no te apetece hacer) […communication (i.e., stating what I like/dislike or I want/don’t want to do)]				
…sentir atracción por tu(s) pareja(s) sexual(es) [...feeling attracted to my sexual partner(s)]				
…el deseo/las ganas que tengas de tener una relación sexual, de hacer algo sexual [...desiring to have a sexual relationship, sexual activity]				
…la concentración, que no estés pensando en otras cosas […my concentration levels, not dwelling on something else]				
…la experiencia/conocimiento que tengas/tengáis […the experience/knowledge I/we have]				
…tu habilidad [...my own ability]				
…la habilidad de tu(s) pareja(s) sexual(es) […my sexual partner’s(partners’) ability]				
…la disposición que tengas a probar cosas nuevas (p.e., innovación, creatividad...) […my disposition to try new things (e.g., innovation, creativity)]				
…que estés tranquila/relajada/a gusto, que nada/nadie te moleste […feeling relaxed/comfortable, unbothered by anything/anyone]				
…tu habilidad para decir a tu(s) pareja(s) sexual(es) cosas bonitas/agradables, o de tu(s) pareja(s) sexual(es) para decirte cosas bonitas/agradables a ti […the ability to express/say pretty/pleasant things to my sexual partner(s) or my sexual partner’s (partners’) ability to do that to me]				
…tu habilidad para decir a tu(s) pareja(s) sexual(es) cosas eróticas, o de tu(s) pareja(s) sexual(es) para decirte cosas eróticas a ti […the ability to express/say erotic things to my sexual partner(s) or my sexual partner’s (partners’) ability to do that to me]				
…que vivas la sexualidad como algo normal/sano/deseable […experiencing sexuality as something normal/healthy/desirable]				
…que no te sientas obligada a hacer algo que no quieres/que no te apetece hacer […not feeling obligated to do something I don’t want/desire to do]				
…“Echarle picante al guiso”, “darle morbo” […making something/someone horny]				
…la compenetración que tengas con tu(s) pareja(s) sexual(es) […having confidence with my sexual partner(s)]				
…el método anticonceptivo que uséis […the contraceptive method used]				
…que ames a tu(s) pareja(s) sexual(es)/que ésta/e(s) te ame(n) […loving my sexual partner(s) or being loved by her/him/them]				
…la confianza/seguridad que tengas en tu(s) pareja(s) sexual(es) […the faith/truth/security with my sexual partner(s)]				
…tu nivel de autoestima […my self-esteem levels]				
…que te sientas cómoda con tu cuerpo/que no sientas vergüenza de tu cuerpo […feeling comfortable with my body/not feeling ashamed]				
…que tú llegues al orgasmo […me reaching climax]				
…que tú y tu(s) pareja(s) sexual(es) lleguéis al orgasmo […me and my partner(s) reaching climax]				
…el sitio donde practicas actividades sexuales (p.e., ruido, tranquilidad, la posibilidad de que te pillen sin querer o queriendo, interrupciones) […the place where the encounter takes place (e.g., noise, peace, the chance of getting caught deliberately or not, interruptions)]				
…que estés muy excitada […being very sexually excited]				
…que sientas que tienes futuro con tu(s) pareja(s) sexual(es), que tu(s) pareja(s) sexual(es) está(n) comprometido/a/s con la relación […feeling that my sexual partner(s) is committed to the relationship]				
…que estés feliz, contenta […feeling contented or happy]				
…tener el mismo nivel de deseo sexual que tu(s) pareja(s) sexual(es) […me and my partner(s) having the same level of desire]				
…que haya respeto, que haya fidelidad […having respect for, faithfulness to each other]				
…que no estés cansada […not being tired]				
…que utilicéis fantasías, juegos, juguetes, etc. […using sexual fantasies, role-playing, erotic toys...]				

12. A continuación te presentamos una lista con diferentes comportamientos sexuales que se pueden realizar individualmente o con tu(s) pareja(s) sexual(es). ¿Cómo de satisfactorios te resultan a ti estos comportamientos? [Below you will find a list of different sexual behaviors that can be performed both individually or with a sexual partner(s). How satisfying are these behaviors to you?]

¿En qué medida influyen los siguientes comportamientos en tu satisfacción sexual? [How much do the following behaviors influence your sexual satisfaction…?]	No lo practico [I do not practice it]	Nada satisfactorio [Not satisfactory at all]	Algo satisfactorio [Somewhat satisfactory]	Bastante satisfatorio [Quite satisfactory]	Muy satisfactorio [Very satisfactory]
Besarse [Kissing]					
Acariciarse [Cuddling]					
Abrazarse [Hugging]					
Lamer o morder suavemente a tu(s) pareja(s) sexual(es), ser lamida o mordida suavemente [Licking or biting my sexual partner(s), being licked/bitten by her/him/them]					
Dar sexo oral (es decir, felación, cunnilingus) [Giving oral sex (i.e., fellatio/cunnilingus)]					
Recibir sexo oral (es decir, cunnilingus) [Receiving oral sex (i.e., cunnilingus)]					
Dar o recibir sexo oral anal (es decir, anilingus) [Giving or receiving anal oral sex (i.e., anilingus)]					
Automasturbación (es decir, autoestimulación manual de los órganos sexuales propios) [Masturbation (i.e., manual self-stimulation of the own genitalia)]					
Heteromasturbación (es decir, estimulación manual de los órganos sexuales de tu(s) pareja(s) sexual(es), o ser estimulada manualmente en los órganos sexuales por esta(s) persona(s), simultáneamente o no) [Hetero-masturbation (i.e., manual stimulation of sexual partner’s(partners’) genitalia, or being manually stimulated on the genitalia by the partner(s), simultaneously or not)]					
Coito vaginal (es decir, introducción pene-vagina) [Vaginal intercourse (i.e., penis-vagina introduction)]					
Coito anal (es decir, introducción pene-ano) [Anal intercourse (i.e., penis-anus introduction)]					
Imaginar/fantasear con otros contextos, situaciones, personas [Using my imagination/fantasies regarding other contexts, situations, people…]					
Poner en práctica alguna fantasía sexual, probarla [Putting into practice any sexual fantasy, practicing it]					
Jugar a la seducción con tu(s) pareja(s) sexual(es) (p.e., ligar, hacer striptease, etc.) [Erotic play, seducing my partner(s) (e.g., flirting, performing a striptease)]					
Acariciar sexualmente el cuerpo de tu(s) pareja(s) sexual(es) sin ropa y sin incluir los órganos sexuales (petting) [Petting]					
Acariciar sexualmente el cuerpo de tu(s) pareja(s) sexual(es) sin ropa, incluyendo los órganos sexuales (heavy petting) [Heavy petting]					
Imitar los movimientos del coito (pseudo-coito) [Pseudo-coitus (i.e., imitate intercourse movements)]					
Usar juguetes sexuales (p.e., balas vibradoras, “satisfier”, anillos vibradores para el pene, estimuladores del clítoris) [Using sex toys (e.g., bullet vibrator, “satisfier”, penis rings, clitoral stimulators)]					
Usar literatura erótica/pornográfica (p.e., novelas, cómics) [Using erotic/pornographic literature (e.g., novels, comics)]					
Usar material visual erótico/pornográfico (p.e., películas, fotografías, vídeos) [Using erotic/pornographic visual media (e.g., movies, photos, videos)]					
Realizar prácticas en las que se retrasa o impide el orgasmo (p.e., sexo tántrico) [Engaging in sexual practices aimed to delay or impede climax (e.g., tantric sex)]					
Realizar prácticas más provocadoras, pero con poco o ningún riesgo (p.e., pellizcos, usar ataduras, pegar cachetes, tapar los ojos) [Engaging in more provocative but non-risky sexual practices (e.g., pinching, using ties, slapping, covering the eyes)]					
Realizar prácticas un poco más agresivas, que requieren de palabras de seguridad (p.e., BDSM) [Engaging in more aggressive sexual practices, ones that require safe-words (e.g., BDSM)]					
Practicar sexo con más de una pareja sexual (p.e., tríos, intercambio de parejas (swinging), orgías, dogging) [Engaging in sex with more than one sexual partner (e.g., threesomes, swinging, orgies, dogging)]					
Realizar prácticas que pueden ser potencialmente arriesgadas (p.e., con desconocidas/os, salas oscuras, ruletas) [Engaging in potentially risky sexual practices (e.g., with unknown people, in dark rooms, sex roulettes)]					

13. ¿Cómo de importante es para ti tener una sexualidad activa y satisfactoria en tu vida? [How important is it for you to have an active and satisfying sexuality in your life?]

☐ Nada importante [Not important at all]
☐ Poco importante, hay otras cosas más importantes para mí [Not very important, there are other things more important to me]
☐ Bastante importante [Quite important]
☐ Muy importante [Very important]
